# Evaluation of phage-based decontamination in respiratory intensive care unit environments using ddPCR and 16S rRNA targeted sequencing techniques

**DOI:** 10.3389/fcimb.2024.1442062

**Published:** 2024-08-19

**Authors:** Yinghan Shi, Weihua Zhang, Lina Li, Wencai Wu, Mengzhe Li, Kun Xiao, Kaifei Wang, Zhaojun Sheng, Fei Xie, Xiuli Wang, Xin Shi, Yigang Tong, Lixin Xie

**Affiliations:** ^1^ College of Pulmonary & Critical Care Medicine, Chinese PLA General Hospital, Beijing, China; ^2^ The First Medical Centre, Chinese PLA General Hospital, Beijing, China; ^3^ Key Laboratory of Biomedical Information Engineering of Ministry of Education, Biomedical Informatics & Genomics Center, School of Life Science and Technology, Xi’an Jiaotong University, Xi’an, Shaanxi, China; ^4^ College of Life Science and Technology, Beijing University of Chemical Technology, Beijing, China

**Keywords:** bacteriophages, hospital-acquired infections, drug-resistant bacterial, targeted metagenomics in pathogen, 16S rRNA

## Abstract

**Background:**

*Klebsiella pneumoniae* is a major cause of hospital-acquired infections (HAIs), primarily spread through environmental contamination in hospitals. The effectiveness of current chemical disinfectants is waning due to emerging resistance, which poses environmental hazards and fosters new resistance in pathogens. Developing environmentally friendly and effective disinfectants against multidrug-resistant organisms is increasingly important.

**Methods:**

This study developed a bacteriophage cocktail targeting two common carbapenem-resistant *Klebsiella pneumoniae* (CRKP) strains, ST11 KL47 and ST11 KL64. The cocktail was used as an adjunctive disinfectant in a hospital’s respiratory intensive care unit (RICU) via ultrasonic nebulization. Digital PCR was used to quantify CRKP levels post-intervention. The microbial community composition was analyzed via 16S rRNA sequencing to assess the intervention’s impact on overall diversity.

**Results:**

The phage cocktail significantly reduced CRKP levels within the first 24 hours post-treatment. While a slight increase in pathogen levels was observed after 24 hours, they remained significantly lower than those treated with conventional disinfectants. 16S rRNA sequencing showed a decrease in the target pathogens’ relative abundance, while overall species diversity remained stable, confirming that phages selectively target CRKP without disrupting ecological balance.

**Discussion:**

The findings highlight the efficacy and safety of phage-based biocleaners as a sustainable alternative to conventional disinfectants. Phages selectively reduce multidrug-resistant pathogens while preserving microbial diversity, making them a promising tool for infection control.

## Introduction

*K. pneumoniae* is a significant cause of Hospital-acquired infections (HAIs), which are predominantly transmitted through contamination of the hospital environment or medical equipment (Odoyo et al., 2023). The risk factors for these infections include environmental contamination, reuse or improper disinfection of medical devices, inadequate hand hygiene practices by healthcare workers, and poor ventilation systems (Peters et al., 2022). Research indicates that while current disinfection measures play a crucial role in controlling the spread of these pathogens, their effectiveness is diminishing due to some pathogens developing resistance to existing disinfectants. Furthermore, frequent use of chemical disinfectants can cause equipment corrosion, harm the environment and health of personnel, and may encourage the development of new resistances in pathogens (Boyce, 2016; Kampf, 2018). Given these challenges, the development of new disinfectants, especially those that are both environmentally friendly and highly effective against multidrug-resistant organisms, has become increasingly urgent.

Bacteriophages are viruses that specifically target bacteria, acting as natural bacterial predators, and offer a unique mechanism of action. Bacteriophage therapy is experiencing a renaissance in response to the escalating global antimicrobial resistance crisis (Salmond and Fineran, 2015; Gordillo Altamirano and Barr, 2019). The historical significance of bacteriophages in combating bacterial infections, dating back almost a century, has resurfaced as a promising solution to address antibiotic-resistant pathogens. With applications ranging from combating antibiotic resistance to food safety and environmental health (Moye et al., 2018; Tie et al., 2018; Nick et al., 2022).

Studies have shown the effectiveness of using atomized bacteriophages to reduce bacterial loads on hard surfaces. Studies by Hussain et al. have demonstrated the efficacy of atomized bacteriophages in reducing *Acinetobacter baumannii* levels on hard surfaces (Ho et al., 2016). Furthermore, the work by Maria D’Accolti et al. explored the combined use of phages and a probiotic-based sanitation system to efficiently remove hospital-acquired infection pathogens from various hard surfaces, showcasing a rapid reduction of argeted pathogens. These studies collectively support the use of atomized bacteriophages as a promising strategy for decontaminating hard surfaces and reducing bacterial levels effectively (D'Accolti et al., 2018).

While the efficacy of bacteriophages as biological disinfectants has been established, their impact on CRKP in clinical settings has yet to be thoroughly investigated. Additionally, further research is needed to explore the impact of bacteriophage intervention on environmental microbial communities to ensure the safety and efficacy of this approach in real-world applications. In this study, we developed a phage cocktail targeting two prevalent *K. pneumoniae* strains, ST11 KL47 and ST11 KL64, found in clinical settings. We utilized these bacteriophages as an adjunctive disinfectant to augment environmental cleaning practices. The efficacy of the phage cocktail aerosol in clearing CRKP from the environment was assessed. The primary objective was to evaluate the impact of the bacteriophage treatment on the clearance of environmentally persistent drug-resistant bacteria and to analyze its effects on the overall microbial community structure. This approach aimed to establish a scientifically sound basis for the broader application of phage therapy in infection control practices, particularly against antibiotic-resistant pathogens.

## Materials and methods

### Setting and environmental sampling

The study was conducted in the respiratory intensive care unit of a hospital in Beijing, China. In addition, this study was approved by the institutional review board of the PLA Hospital (309202305011312). To assess the baseline microbial community composition and the molecular characteristics of *K.pneumoniae* on environmental surfaces in the Respiratory Intensive Care Unit (RICU) prior to any phage or chemical disinfectant intervention, bedside environmental samples were collected from patients diagnosed with CRKP infection(n=9). For *K.pneumoniae* infection diagnosis, Metagenomic next-generation sequencing (mNGS) pipeline was performed on six out of these patients, in brief, mNGS and hybridization capture-based targeted mNGS were used to detectpathogens. Approximately 1 mL of sample was centrifuged at 12,000 g for 5 minutes to separate pathogens and human cells. The precipitate was treated with Benzonase and Tween 20, followed by DNA extraction using the QIAamp UCP Pathogen Mini Kit. DNA concentrations were measured with a Qubit 4.0. Libraries prepared from the DNA were sequenced on the NextSeq 550 platform (Illumina). For targeted NGS, libraries were enriched with microbial probes and sequenced data filtered to remove unsuitable reads. Species-level microbial identification was performed using an NCBI database. While the remaining three were identified via microbiological culture of respiratory samples.

This initial sampling was conducted before the application of chlorinated disinfectants or bacteriophage-based sanitation methods. Surface swabs were taken from areas frequently contacted by staff and patients, including floors, bed linens, bed frames and bedside tables, with a total of 36 environmental hard surface samples, each covering an area of 100 square centimeters. These swabs were preserved in phosphate-buffered saline (PBS) and transported to the laboratory in a chilled condition and processed within 2 hours of collection for subsequent DNA extraction and microbial culture.

### CRKP isolates and identification

Each swab will be streaked onto MacConkey agar plates. *K. pneumoniae* was identified by standard methods and confirmed using the “BD Phoenix Automated Microbiology System”. Antimicrobial drug susceptibility testing was performed using the BD system and the minimal inhibitory concentration (MIC) of imipenem and meropenemresults were interpreted using the Clinical and Laboratory Standards Institute 2016. The resistant breakpoint of CRKP to imipenem and meropenem was defined as a MIC > 4 μg/mL.

### Source and selection of active bacteriophage

In this study, bacteriophages were isolated from sewage using the double-layer agar plate method. Briefly, untreated wastewater samples were centrifuged and then filtered through a 0.22µm filter (Millipore, USA) to remove bacteria and other particles. A volume of 500 µL of the filtrate was co-cultured with 500 µL of *K. pneumoniae* (OD600 = 0.8) in 5 mL of LB medium, and incubated overnight at 37°C with shaking. The mixed culture was then centrifuged at 12,000 x g for 2 minutes and the supernatant containing the phages was collected by filtration through a 0.22µm filter. Then, CRKP isolated from the ward environment samples was added to soft LB agar (0.5%, haibo, qingdao) and then poured onto a regular LB agarplate. Subsequently, 2 µl aliquots of different phage solutions (approximately 10^8^ plaques forming units (PFUs)) were spotted on the bacterialplate. Finally, the plates were incubated at 37 °C for 24 h. Generation of a clearance zone surrounding the phage inoculation spots indicates that the CRKP host was susceptible to the inoculated phage.

### Genomic identification of *K. pneumoniae*

The *K. pneumoniae* strains isolated from environmental samples were cultured in LB liquid medium to the logarithmic growth stage. The genomic DNA of *K. pneumoniae* isolates was extracted by Bacterial Genomic DNA Extraction kit (Beijing Solarbio Science & Technology Co., Ltd.) and whole genome sequencing of 16 K*. pneumoniae* isolates obtained from environmental samples was performed using Illumina HiSeq-150. The raw sequencing reads of *K. pneumoniae* was assembled using SPAdes v3.13.0. The MLST, resistance, and virulence of *K. pneumoniae* was predicted using the integrated tool Pathogenwatch(https://pathogen.watch/).

### Environmental decontamination by phage-containing aerosols

In this study, we evaluated the efficacy of bacteriophage disinfectants compared to chemical disinfectants for decontaminating environmental surfaces contaminated with *K. pneumoniae*, in a hospital setting. Two wards, housing patients infected with *K. pneumoniae*, were elected for environmental cleaning.

Initial environmental sampling (T1) was performed prior to the application of any disinfectant to establish a baseline pathogen load. Then use a chemical disinfectant, and a second sampling (T2) was taken six hours later to evaluate the immediate efficacy of the chemical agents. Subsequently, apart from chemical disinfectants, a bacteriophage-based disinfectant was additionally used. The phages solution was aerosolized using an ultrasonic nebulizer, with the phage concentration in the ultrasonic humidifier set at 10^8^ PFU (plaque-forming units) per milliliter, and approximately 500 mL utilized per room. Further samples were collected at 24 (T3), 48 (T4), and 72 (T5) hours post-application of the bacteriophage disinfectant, enabling the assessment of its long-term disinfection performance.

The hospital daily decontamination schedule was not changed during the bacteriophage decontamination process. For environmental decontamination, the phage stock solution was diluted in normal saline, and the phage aerosols were then generated with an ultrasonic humidifier (Rimei Electronic Technology Co. LTD) for 20 min, to ensure homogenization distribution of the phage aerosols throughout the ward.Briefly, we attached a disposable sterile sampling bag to the outlet of the ultrasonic nebulizer to collect the aerosolized phage cocktail. After initiating the machine and allowing the nebulization process to complete, we used a pipette to aspirate the condensed phage cocktail droplets from the sterile sampling bag. The collected droplets were then mixed thoroughly, serially diluted, and the phage titer was determined using the double-layer agar method.

### DNA extraction, PCR amplification

The genomic DNA was extracted by SDS method, and the DNA concentration and purity were detected by agarose gel electrophoresis. According to the concentration results, the DNA was diluted to l ng/μL with sterile water. Specific primers were used for PCR amplification using diluted genomic DNA as a template. The amplification process consisted of predenaturation at 98°C for 1 min, 30 cycles (denaturation at 98°C for 10 s, annealing at 50°C for 30 s, extension at 72°C for 30 s), and finally extension at 72°C for 5 min. Then the mixed PCR products were purified. Samples were divided into room, position, time, and sample types.

### Droplet digital PCR analysis and amplicon sequencing

The ddPCR assays were conducted to quantify *K. pneumoniae* DNA in the samples, using specific primers and probes designed for *K. pneumoniae. The* ddPCR assay was performed in a reaction volume of 20μL using a commercial ddPCR kit (Xinyi, Beijing, China). Twenty microliters of PCR mixture,50μL Droplet generation oil and 5μL sealant were mixed, and droplet generation was performed using the test instrument MicroDrop -100A. The droplet emulsion was thermally cycled in the following conditions: predenaturing at 95°C for 10 min, 45 cycles of PCR at 95°C for 30 s, and at 60°C for 1 min, and the test instrument was cooled at 12°C for 5 min, and then, the reaction was finished. The ddPCR system partitioned the DNA samples into approximately 20,000 nanoliter-sized droplets, with PCR amplification occurring in each droplet independently. The PCR reaction plate was placed in the MicroDrop-100B biochip reader for detection. Analyze data results using QuantDrop data analysis software.

To assess the broader impact on the microbial community, 16S rRNA gene sequencing was performed on the same samples. The V3-V4 region of the 16S rRNA gene was amplified using universal primers. The TruSeq DNA PCR-Free Sample preparation Kit was used to construct the library from the purified amplified fragments. After qualified, the library was sequenced using Illumina NovaSeq sequencing platform.

### 16S rRNA reads sequencing data processing

The original data obtained by sequencing was spliced and filtered to remove interference data and obtain effective data that can be used for subsequent analysis. Then, the valid data were grouped into Operational Taxonomic Units (OTUs) with 97% consistency, and the OTUs sequence was compared with the SSUrRNA database for annotation at kingdom, phylum, class, order, family, and genus levels. The species composition of each sample is counted. QIIME version 2 was used to estimate alpha and beta diversity (Bolyen et al., 2019). OTU abundances were used to calculate the alpha diversity metrics, including OTU richness (unique OTUs), ChaoI richness estimation, and Shannon’s diversity indices. For overall comparison of significant differences among bacterial communities (i.e., beta diversity), principal coordinates analysis (PCoA) was performed.

## Results

### CRKP isolates and their phage susceptibility

**Figure 1:**
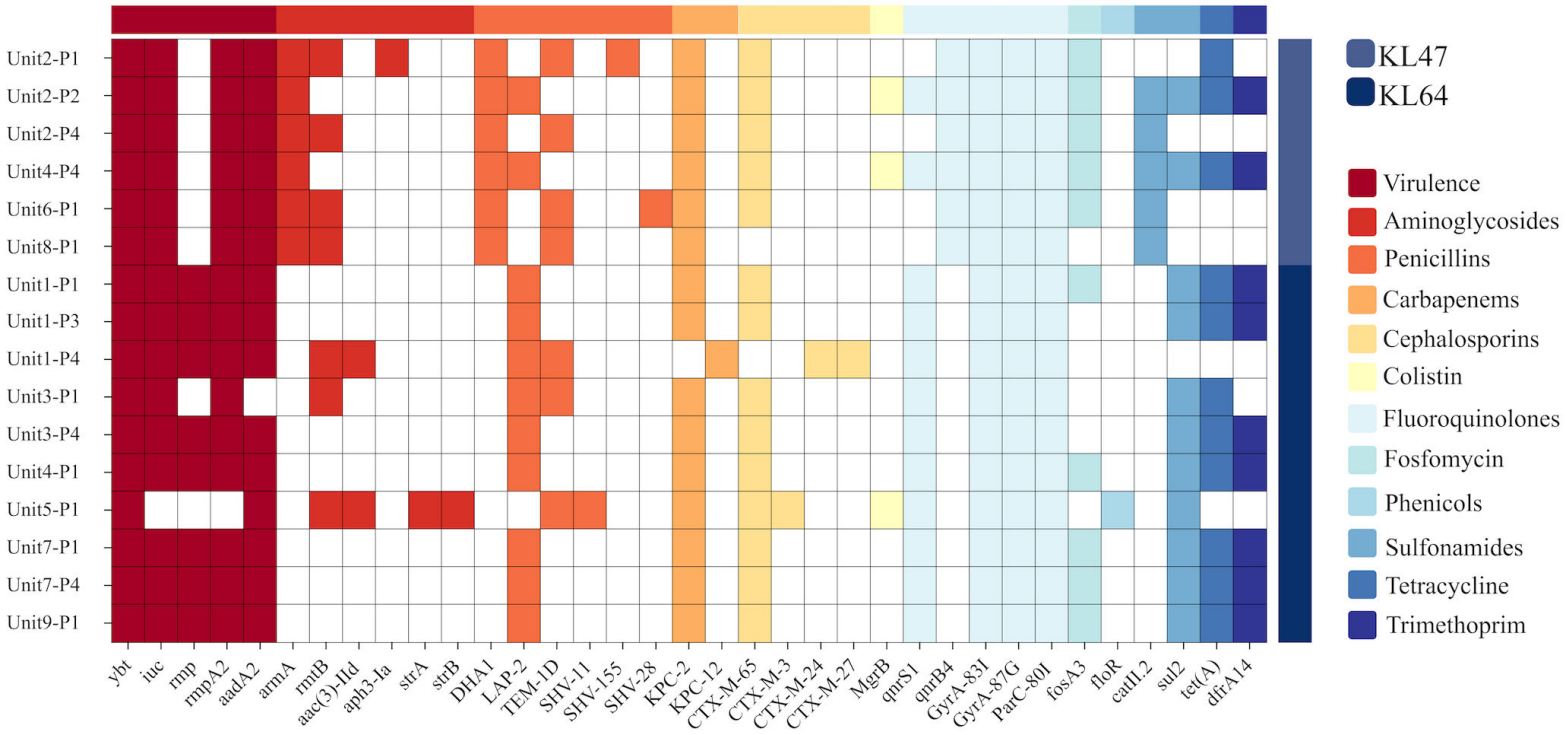
Genotypic characterisation and analysis of 16 K.pneumoniae isolates. This figure displays the distribution of prevalent sequence types ST11 KL64 and ST11 KL47, and account of virulence factors and antibiotic resistance genes. Virulence genes include ybt, iuc, rmpA and rmpA2, which are linked to the mucoid phenotype. Resistance genes span multiple antibiotic classes: Aminoglycosides (aadA2, armA), Penicillins(TEM-1D, SHV-11, SHV-15, SHV-28), Carbapenems(KPC-2, KPC-12), Cephalosporins (CTX-M-65, CTX-M-3, CTX-M-24, CTX-M-27), and Fluoroquinolone(GyrA and ParC).

*K. pneumoniae* was cultured in 16 out of 36 environmental samples taken before the disinfection procedure (**Table 1**). The study findings indicated a universal presence of *K. pneumoniae* on the floors near all patient bed units. Pillowcases also showed a significant presence of the pathogen, with five out of nine (n=5) testing positive. In contrast, bed rails and bedside tables exhibited lower detection rates. These isolates were identified as CRKP using the BD Phoenix Automated Microbiology System. The whole genome sequencing results indicate that the *K. pneumoniae* isolates belong to the ST11 KL47 (n=6) and ST11 KL64 (n=10) sequence types (**Figure 1**).

**Table 1:** Microbial culture results for K. pneumoniae in samples collected from various sites adjacent to bedside units in hospital wards housing nine infected patients.

	Unit1	Unit2	Unit3	Unit4	Unit5	Unit6	Unit7	Unit8	Unit9
P1	+	+	+	+	+	+	+	+	+
P2	−	+	−	−	−	−	−	−	−
P3	+	−	−	−	−	−	−	−	−
P4	+	+	+	+	−	−	+	−	−

The table is structured with the horizontal axis representing the nine bedside units, and the vertical axis indicating different locations within each room: P1 for the floor, P2 for the bedside table, P3 for the bed rail, and P4 for the bedsheet. ‘-’ indicates no colonies were cultured, while ‘+’ indicates presence of colonies.

From each of these 16 culture dishes, one bacterial strain was selected for phage typing. The isolated strains were susceptible to *K. pneumoniae* type ST11 KL47 or ST11 KL64 as host infection (**Table 2**).The phages with the best lytic performance were composed into a phage cocktail, consisting of one phage against *K. pneumoniae* type KL47 and two phages against *K. pneumoniae* type KL64 (**Supplementary Figure 1**).

**Table 2:** Phage sensitivity matrix for K. pneumoniae colonies cultured from various locations within different rooms, assessed using the double-layer agar spot test.

Bacteria Phage	Unit1-P1	Unit1-P3	Unit1-P4	Unit2-P1	Unit2-P2	Unit2-P4	Unit3-P1	Unit3-P4	Unit4-P1	Unit4-P4	Unit5-P1	Unit6-P1	Unit7-P1	Unit7-P4	Unit8-P1	Unit9-P1
PCCM_KpP42	−	−	−	+	+	+	−	−	−	+	−	+	−	−	+	−
PCCM_KpP216	−	−	−	+	+	+	−	−	−	+	−	+	−	−	+	−
PCCM_KpP416	−	−	−	+	+	+	−	−	−	+	−	+	−	−	+	−
PCCM_KpP24	+	+	+	−	−	−	+	+	+	−	+	−	+	+	−	+
PCCM_KpP22	+	+	+	−	−	−	+	+	+	−	+	−	+	+	−	+
PCCM_KpP112	+	+	+	−	−	−	+	+	+	−	+	−	+	+	−	+
PCCM_KpP1119	+	+	+	−	−	−	+	+	+	−	+	−	+	+	−	+
PCCM_KpP27X	+	+	+	−	−	−	+	+	+	−	+	−	+	+	−	+
PCCM_KpP1172	+	+	+	−	−	−	+	+	+	−	+	−	+	+	−	+

The table is structured with the horizontal axis listing the K. pneumoniae colonies identified by room and specific location, while the vertical axis denotes the phages used for typing, where ‘-’ indicates phage insensitivity and ‘+’ denotes phage sensitivity.

The phage stock solution with a titer of 10^8^PFU/mL was introduced into the ultrasonic atomizer. After undergoing ultrasonic atomization, the titer of the recovered phage dropped to 10^7^PFU/mL. This one-log decrease in titer suggests that the phage was adversely affected by the physical processes, such as heat generation or vibrations, associated with the ultrasonic atomization (**Supplementary Figure 2**).

### *K. pneumoniae* quantification

**Figure 2:**
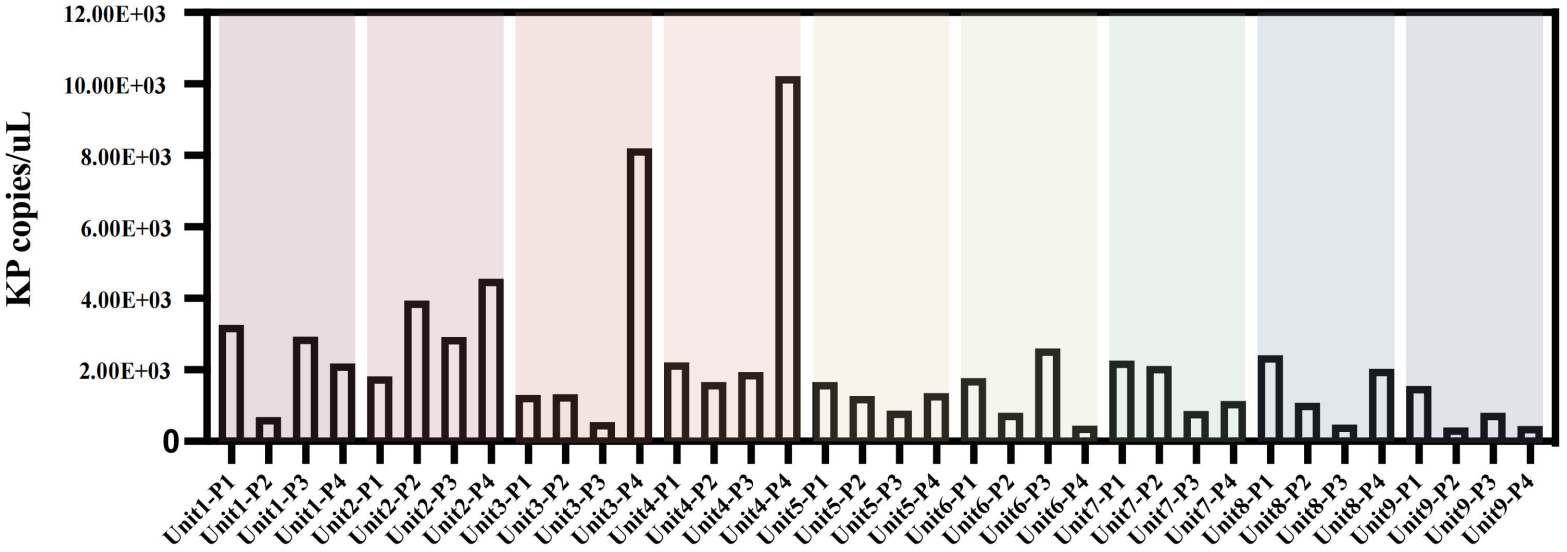
Copies of K.pneumoniae pathogens in bedside environmental samples from 9 patients without disinfection. Unit represents different bedside elements: P1 for the floor, P2 for the bedside table, P3 for the bed rail, and P4 for the bedsheet.

Although only 16 samples were culturably positive, we detected *K. pneumoniae* at different molecular levels in all 36 samples by ddpcr.The quantitative results of *K. pneumoniae* in each sample are shown in the figure (**Figure 2**).The findings revealed that bed linens exhibited the highest total copy numbers of the pathogen (3.03×10^4^ copies/uL), indicating significant reservoirs of the organism in these locations, the floors also demonstrated a considerable presence (1.69×10^4^ copies/uL), while lower copy numbers were observed on bed frames (1.33×10^4^ copies/uL) and bedside tables (1.34×10^4^ copies/uL) (**Supplementary Table 1**).These findings highlight that floors and bed linen are frequent areas of contact between health care workers and patients in the healthcare environment, and these areas can be an important route for pathogen transmission, especially bed linens, which are in direct contact with the face and respiratory tract of patients, and can easily become a source of cross-infection.

### 16S rRNA sequencing analysis: microbial community composition and diversity

**Figure 3:**
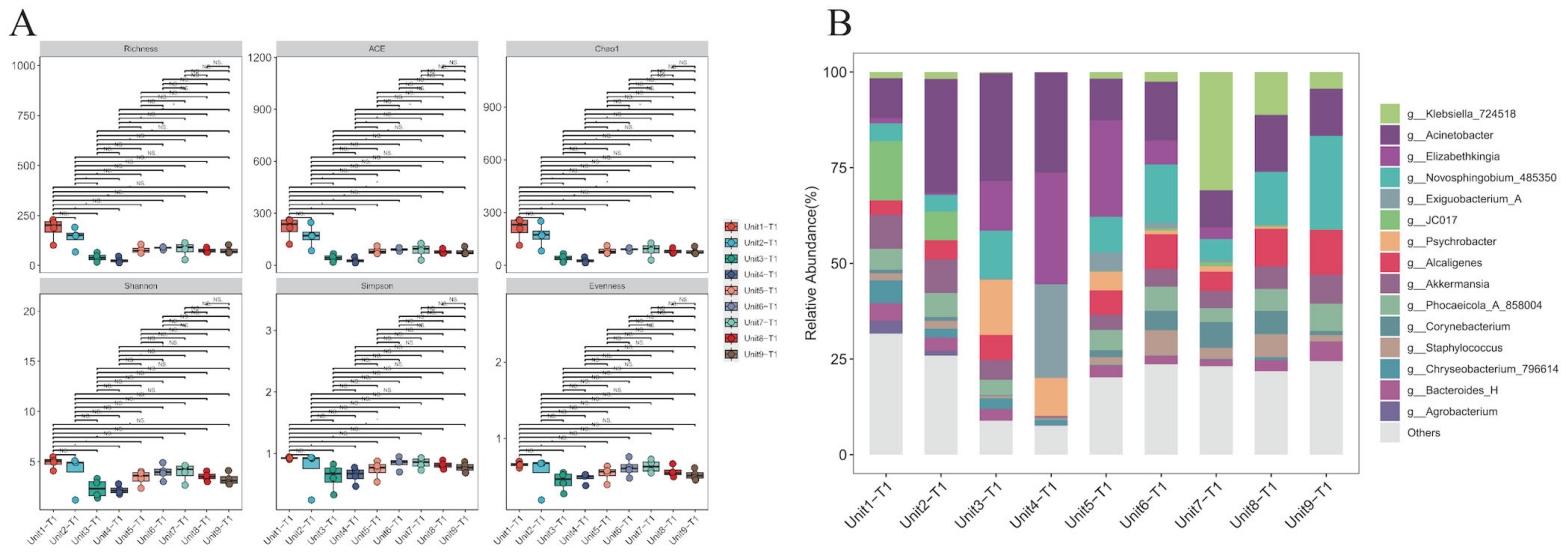
Species diversity and species composition of environmental hard surface samples without disinfection. **(A)** Analysis of alpha diversity of samples on hard surfaces near the bedside of K.pneumoniae infected patients without prior disinfection. **(B)** Composition of microbial genera on hard surfaces adjacent to the bedsides of patients infected with K. pneumoniae without prior disinfection. Common hospital pathogens include Klebsiella, Acinetobacter, Staphylococcus, and Elizabethkingia. Environmental symbiotic bacteria or opportunistic bacteria include Akkermansia, Corynebacterium, and Bacteroides.

Microbial community composition and diversity analysis in environmental samples from the RICU prior to disinfection were conducted using 16S rRNA sequencing. The α-diversity analysis across different bedside units shows similar microbial community compositions (P > 0.05), reflecting the stability of microbial communities within the ward environment (**Figure 3A**). In the hard surface environment samples from unsterilized bedsheets, we counted the top 15 species. The results show that, in addition to *Klebsiella*, common nosocomial pathogens such as *Acinetobacter* (17%) and *Elizabethkingia* (8%) were observed to hold dominant positions across all facilities surveyed. In contrast, *Staphylococcus* accounted for a comparatively smaller proportion of the microbial community (2.6%). Furthermore, the presence of *Akkermansia* (5.5%)—a genus associated with environmental symbiosis or opportunistic infections—as well as *Corynebacterium* (2.5%) and *Bacteroides* (2.9%), indicates that although *Klebsiella* is widespread, it does not dominate the microbial ecosystem (**Figure 3B**).

### Effect of bacteriophage aerosol on environmental microorganisms

**Figure 4:**
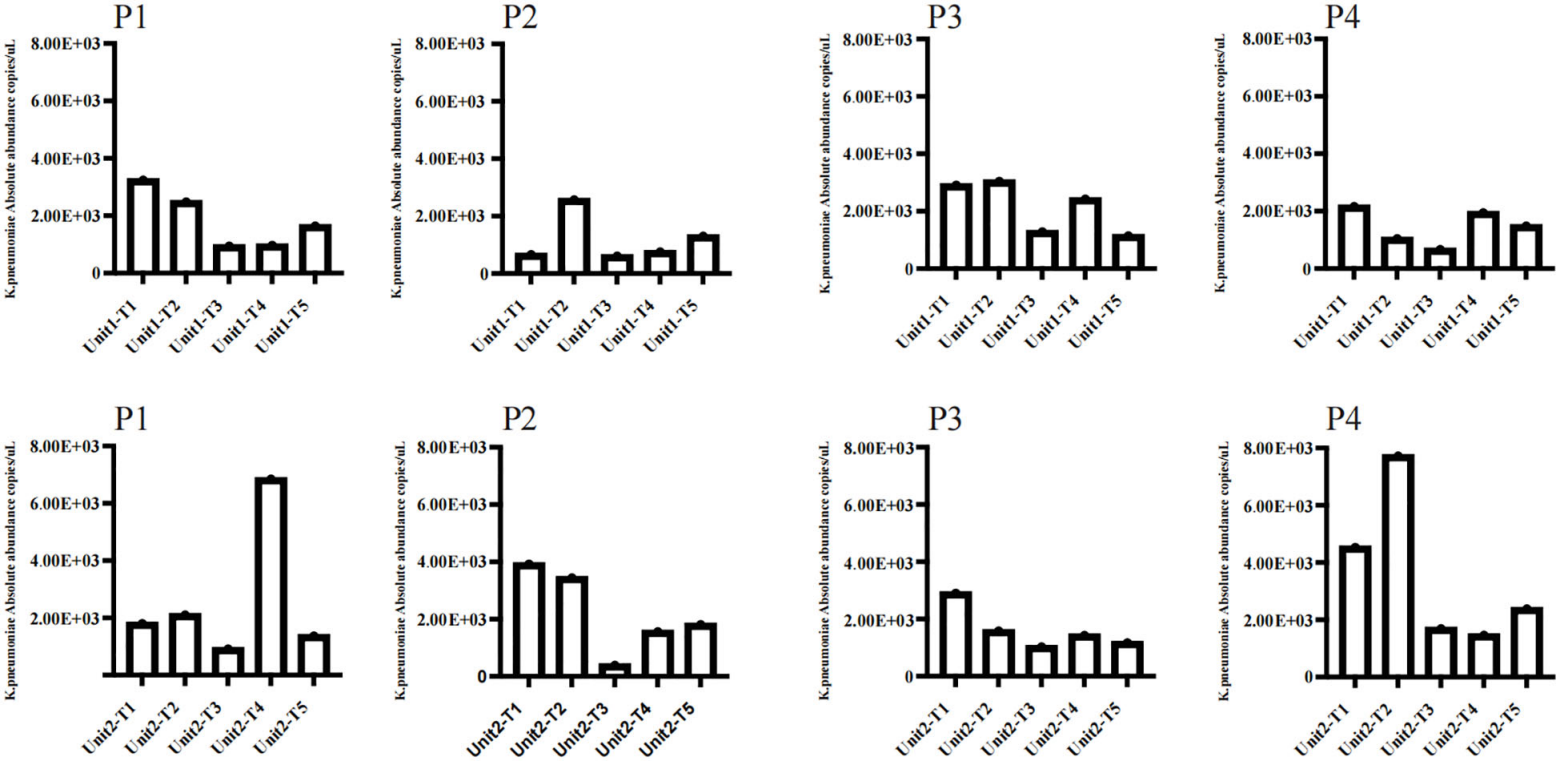
Employed ddPCR to quantitatively assess the genomic copy numbers of *K. pneumoniae* in two rooms disinfected with phage cocktails at five distinct time intervals. The sampling points included P1 for the floor, P2 for the bedside table, P3 for the bed rail, and P4 for the bedsheet. The time intervals were as follows: (T1) Before using any disinfectant; (T2) 6 hours after disinfection with chemical disinfectants; (T3) 24 hours after disinfection with phage cocktails; (T4) 48 hours after disinfection with phage cocktails; (T5) 72 hours after disinfection with phage cocktails.

In this investigation, ddPCR was employed to quantitatively analyse the genomic copies of *K. pneumoniae* at five distinct time intervals, to compare the immediate and sustained impacts of chemical and bacteriophage disinfectants (**Figure 4**). There were no significant differences in the absolute abundance of *K. pneumoniae* before and 6 h after chemical disinfection, which were respectively 8.97×10^3^ and 9.13×10^3^ copies/μL in Unit 1, and 1.31×10^4^ and 1.48×10^4^ μL in Unit 2.In contrast, a significant reduction in *K. pneumoniae* load was observed in samples treated with additional bacteriophage disinfectant for 24 hours compared to those treated with chemical disinfectants, and the absolute abundance of *K. pneumoniae* decreased to 3.49×10^3^ and 4.02×10^3^ copies/μL in Unit 1 and 2, respectively.Further assessments at 48 and 72 hours post-bacteriophage application showed that the majority of samples maintained low pathogen levels, with only slight increases.The absolute abundance of *K. pneumoniae* in unit 1 and Unit 2 was 6.07×10^3^ and 1.12×10^4^ copies/μL after 48 hours of application of phage cocktail, and after 72 hours in unit 1 and Unit 2 was 5.55×10^3^ and 6.7×10^3^ copies/μL. Notably, a sharp increase in pathogen copy numbers was observed 48 hours post-bacteriophage application on the floor near the second bed unit, potentially linked to high-risk procedures such as suctioning, which had been conducted in the vicinity.

**Figure 5:**
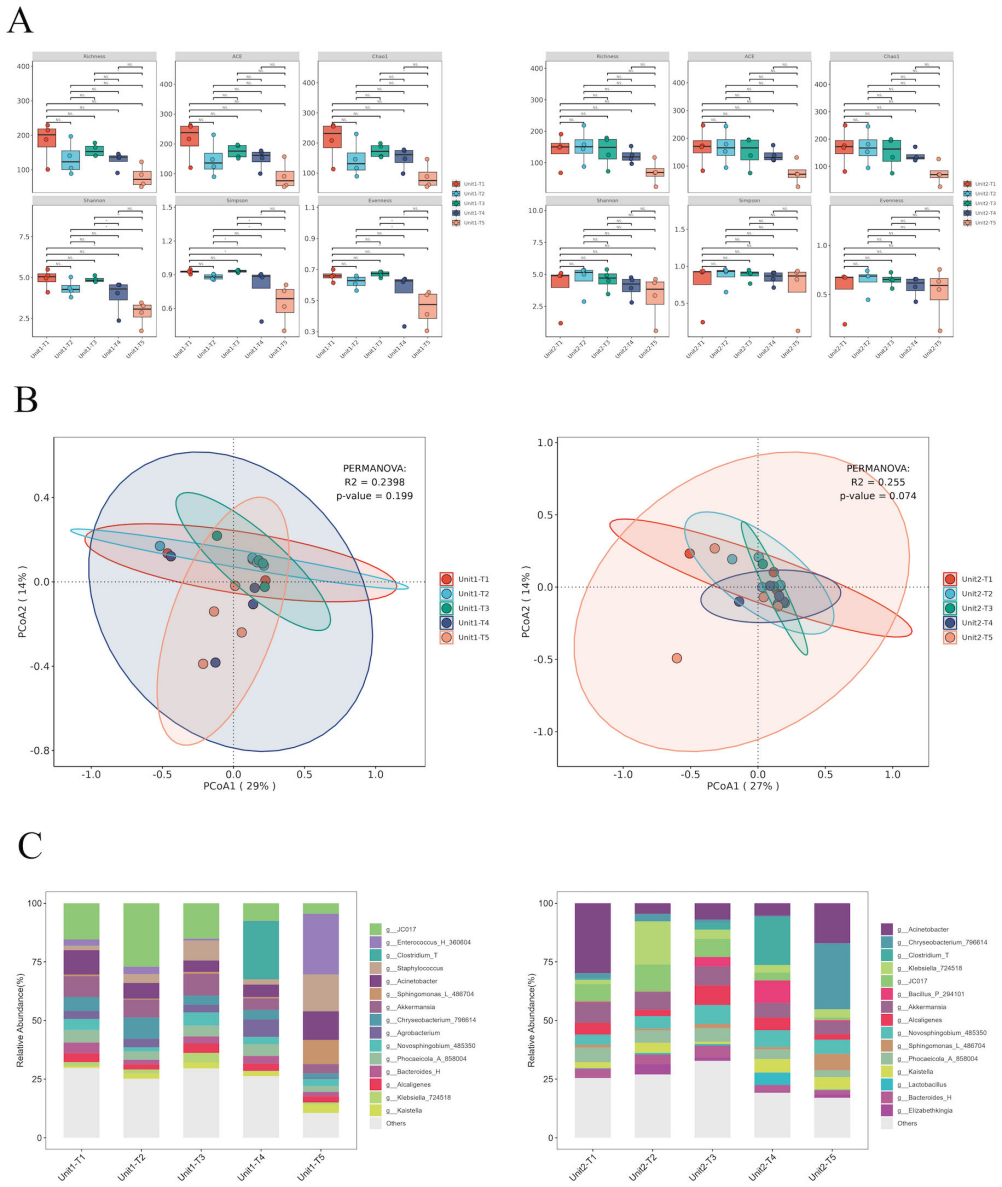
Species diversity and species composition of environmental hard surface samples under different treatments of Unit-1 and Unit-2: **(A)** Analysis of species α diversity in environmental hard surface samples before and after phage cocktail intervention. **(B)** Analysis of species β diversity in environmental hard surface samples before and after phage cocktail intervention. **(C)** Temporal changes in the composition of microbial genera on hard surfaces within two hospital rooms treated with phage cocktail disinfectants. T1: Before using any disinfectant, T2: 6 hours after disinfection with chemical disinfectants, T3: 24 hours after disinfection with phage cocktails, T4: 48 hours after disinfection with phage cocktails, T5: 72 hours after disinfection with phage cocktails.

To assess the impact of phage treatment on the broader microbial community, 16S rRNA sequencing was performed on the same environmental sample. Our analysis of α and β diversity indices pre- and post-bacteriophage intervention on hard surface samples in a healthcare setting revealed a remarkable stability in microbial community structure (**Figures 5A, B**). This observation underscores the host-specific action of bacteriophages, which selectively target specific pathogens without disturbing the overall microbial equilibrium (P >0.05).

Initially, *Klebsiella* constituted 6.5% and 7.3% of the microbial community. Post-application of chemical disinfectants, the relative abundance of *Klebsiella* increased marginally to 6.8% in one sample, but alarmingly to 73% in another, suggesting potential disparities in disinfectant application or microbial resistance. Conversely, bacteriophage cocktail treatments demonstrated substantial efficacy. After 24 hours, the prevalence of *Klebsiella* turn into 16% and 15%. This reduction persisted at 48 hours with further declines to 1.2% and 12%, albeit a slight resurgence was observed at 72 hours (3.3% and 14%), possibly due to phage titre decrease or bacterial adaptation. These findings underscore the potential of phage-based disinfectants in significantly reducing *Klebsiella* load, highlighting their advantages over traditional chemical disinfectants, especially given the variable efficacy and potential for resistance development observed with the latter (**Figures 5C**).

## Discussion

Hospital environmental contamination is a key factor in healthcare-associated infections (HAIs) (Cruz-López et al., 2023; Freier et al., 2023). Traditionally, pathogen detection in environmental samples relies on microbial culture and colony counting (Song et al., 2018). This method is limited in rapidly and accurately identifying specific pathogens. In our study, using both microbial culture and ddPCR to detect *K. pneumoniae* on untreated ward surfaces, we found only 16 positive samples via culture, whereas ddPCR detected *K. pneumoniae* DNA in all samples. This highlights ddPCR’s sensitivity in detecting low-abundance or non-viable bacteria.

Regular monitoring of *K. pneumoniae* in hospital environments is crucial for preventing HAIs. In China, the ST11 CRKP clone, which has undergone virulence evolution, is prevalent in clinical settings (Wang et al., 2024). Our study detected the ST11KL64 and ST11KL47 strains of CRKP on hard surfaces in a respiratory intensive care unit, indicating their potential role in transmitting HAIs due to their survival capabilities. As antibiotic resistance worsens, traditional chemical disinfectants face increasing challenges in controlling HAIs. These chemicals can disrupt microbial communities, harm beneficial microbes, and promote resistant microbial populations (Nordholt et al., 2021; Romero-Fierro et al., 2021). Recently, phage therapy, a targeted biological control strategy, has gained renewed interest for treating infections caused by resistant bacteria.The use of bacteriophages as biological disinfectants in hospital environments has shown promising feasibility, and the safety of using bacteriophages as biological disinfectants in hospital environments is well-documented and supported by multiple aspects (Cisek et al., 2017). Firstly, bacteriophages are naturally occurring entities found widely in the environment and within the human body, where they coexist without causing significant adverse effects (Lin et al., 2017). Their high specificity means they target only particular bacterial strains, thus sparing human cells and beneficial microbiota from harm. This minimizes collateral damage to the host’s microbiome, which is an important consideration in maintaining overall health. Moreover, bacteriophages biodegrade after completing their lifecycle, leaving no long-term residues in the environment. This characteristic reduces the ecological impact of their use in disinfection processes.

This study investigates the potential of using phage cocktails to clean clinical environments and analyzes changes in environmental microbial communities before and after intervention via 16S rRNA amplicon sequencing. Compared to traditional chemical disinfectants, phages, as biological agents, offer distinct advantages in the cleaning process: they specifically target and eliminate bacteria, reducing harm to beneficial microbes and thus maintaining microbial balance. They can also keep target pathogens at low levels over extended periods. Additionally, employing phages reduces reliance on antibiotics and decreases the risk of developing antibiotic resistance. These characteristics make phage cleaning methods especially important and promising in modern medical settings.

This study has certain limitations. Firstly, the short duration of the study may not fully capture the long-term efficacy and potential rebound of pathogens. While we employed the latest ddPCR technology to enhance the sensitivity of pathogen detection, logistical and resource constraints prevented us from implementing extended monitoring in this study. This limitation may have led to an incomplete observation of pathogen dynamics over a longer period. Additionally, although we demonstrated the short-term efficacy of bacteriophages, the number of pathogens began to rebound within 24 hours post-treatment. This suggests that the high specificity of bacteriophages may only target specific host bacteria. Over time, some bacteria may develop resistance to the phages through mutations in phage receptor sites. These resistant strains can survive the initial phage attack and begin to repopulate, causing a rebound in bacterial numbers. Future research needs to explore how to optimize the composition of phage cocktails to cover a broader range of pathogen species and improve their persistence in complex hospital settings.

Finally, while bacteriophages show potential as environmental disinfectants, a number of factors need to be considered before practical application, including bacteriophage preparation, stability, cost-effectiveness, and regulatory issues. Future studies should aim to address these practical issues while evaluating the universality and efficiency of phages in different types of hospital Settings. By optimizing phage preparations and applications, a new hospital disinfection strategy that is both environmentally friendly and efficient is expected to be realized.

## Data Availability

The data presented in the study are deposited in the online repository. The names of the 16S rRNA repository/repositories and accession number(s) can be found below: https://www.ncbi.nlm.nih.gov/genbank/, BioProject ID: PRJNA1090370. The names of the Targeted pathogen detection repository/repositories and accession number(s) can be found below https://ngdc.cncb.ac.cn/sso/login, BioProject ID: PRJNA1122928.
