# Supplementary figures and images for: Evaluation of phage-based decontamination in respiratory intensive care unit environments using ddPCR and 16S rRNA targeted sequencing techniques
Source: Front Cell Infect Microbiol. 2024 Aug 19;14:1442062. doi: 10.3389/fcimb.2024.1442062 (PMC11366697; doi:10.3389/fcimb.2024.1442062)

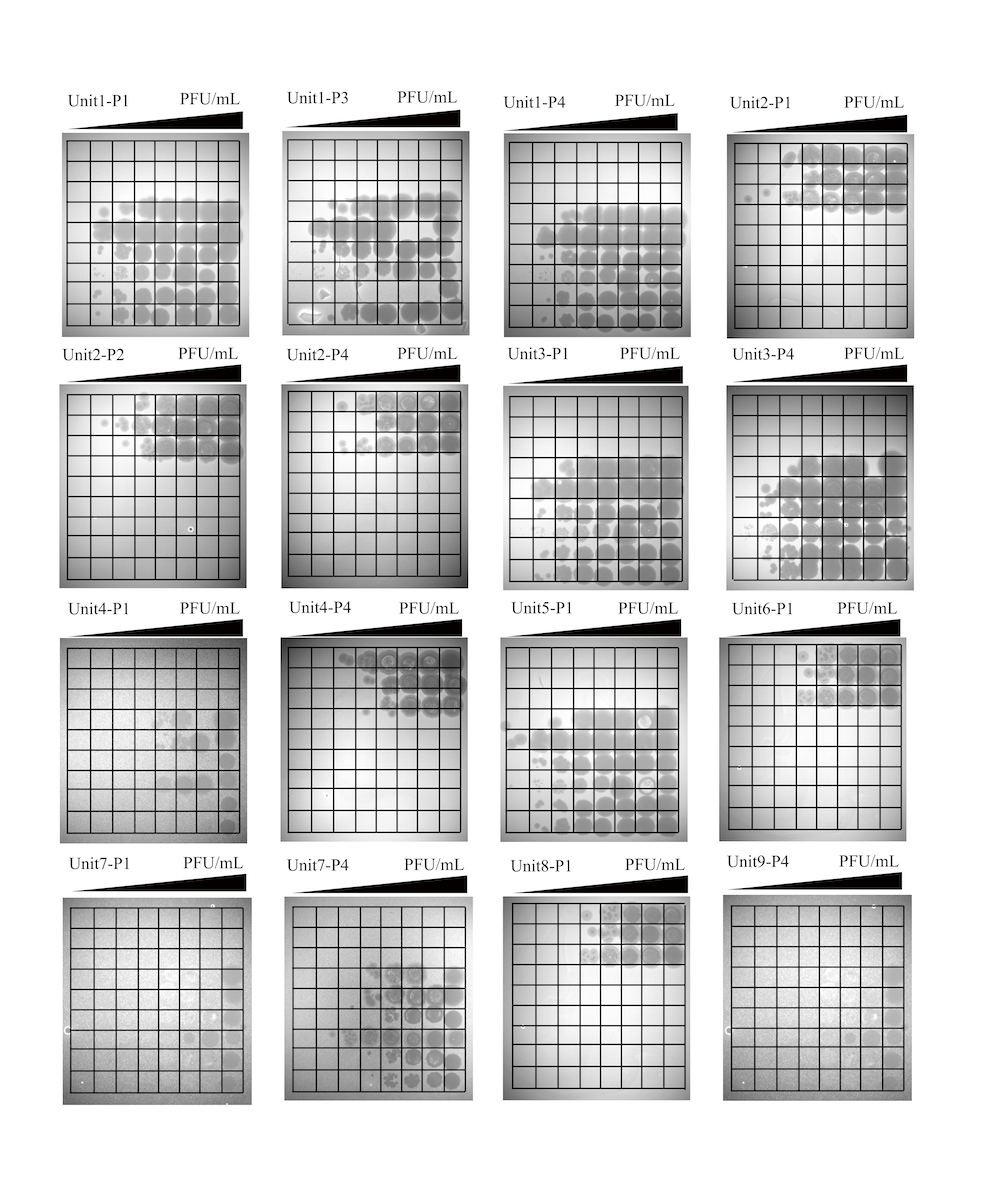

Supplement: Supplementary file 3 [file Image1.tif]

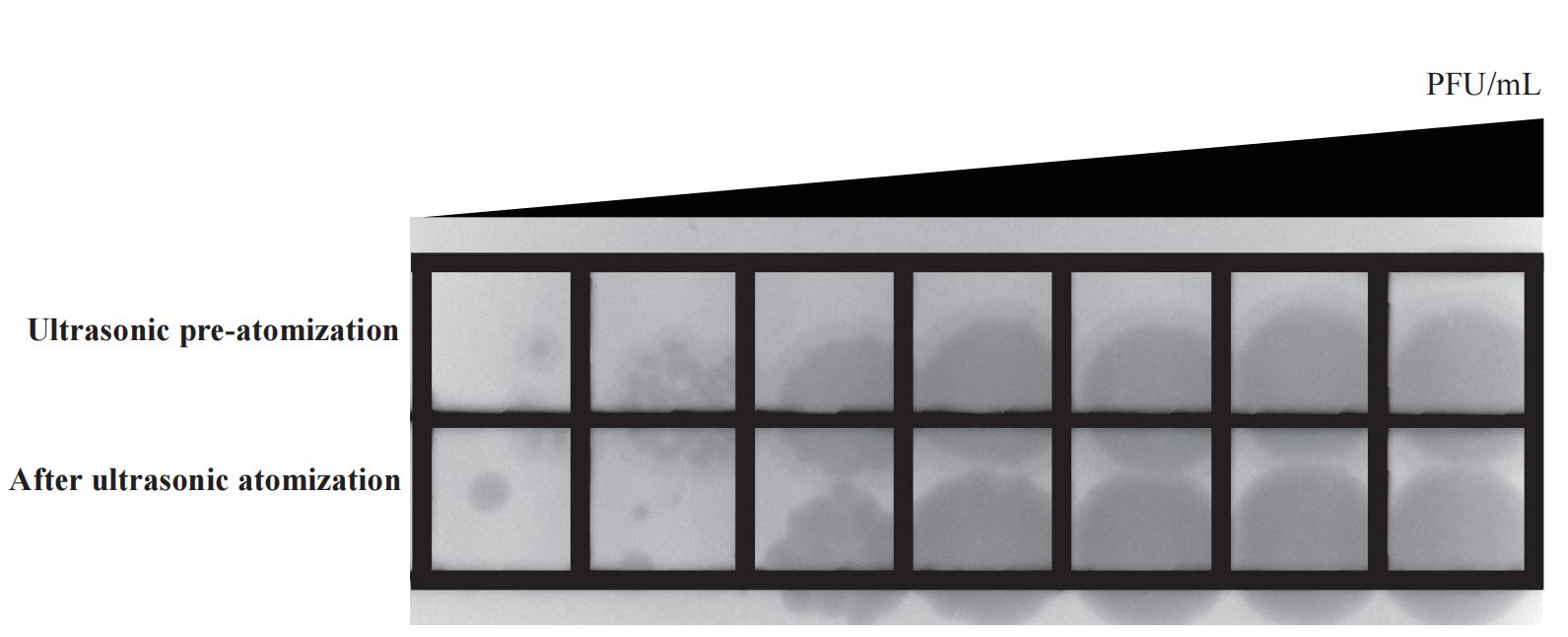

Supplement: Supplementary file 4 [file Image2.tif]
